# Cross-cultural validation and reference norms for the DCDDaily-Q questionnaire Chinese version (DCDDaily-Q-CN): evaluating children’s motor performance in activities of daily living

**DOI:** 10.3389/fpubh.2024.1522816

**Published:** 2025-01-09

**Authors:** Meihuan Huang, Yujun Zhan, Haishan Zhou, Ping Song, Yanping Fan, Yang Lu, Yijing Chen, Zhen Lv, Qing Liu, Guojun Yun, Jianguo Cao

**Affiliations:** ^1^Department of Rehabilitation Medicine, Shenzhen Children’s Hospital, Shenzhen, Guangdong, China; ^2^Department of Pediatric Neurorehabilitation, Shenzhen Dapeng New District Maternity and Child Health Hospital, Shenzhen, Guangdong, China; ^3^Department of Child Healthcare, Pingshan District Maternal and Child Healthcare Hospital of Shenzhen, Shenzhen, Guangdong, China; ^4^Child Healthcare Department, Shenzhen Maternity & Child Healthcare Hospital Co-construction Hospital, Shenzhen, Shenzhen, Guangdong, China

**Keywords:** DCDDailly-Q Chinese version, developmental coordination disorder, normative data, activities of daily life (ADL), children

## Abstract

**Background:**

The DCDDaily-questionnaire (DCDDaliy-Q) evaluates children’s performance and participation in motor-based activities of daily living (ADLs), meeting diagnostic criterion B for developmental coordination disorder (DCD). Currently, there are no Chinese translations or growth references available. Thus, this study aimed to culturally adapt, validate, and establish reference norms for the DCDDaily-Q in Chinese children.

**Methods:**

The original scale was translated and culturally adapted into Chinese (DCDDaily-Q-CN) following international guidelines. Normative data of typically developing children (*n* = 1936, aged 5–10) were gathered from 14 randomly chosen mainstream schools in a large migrant city. Thirty children (aged 5–10 years) diagnosed with DCD were recruited through clinical referrals, and a matched control group (*n* = 30) was randomly selected from the reference group. Reference norms with growth curves and psychometric properties were analyzed.

**Results:**

Sex-specific growth curves with percentiles and cut-off values of the DCDDaily-Q-CN in children aged 5–10 years were established. The instrument demonstrated excellent internal consistency across the total and the three subscales (Cronbach’s alpha = 0.83–0.91). The confirmatory factor analysis showed a good fit for the original three-factor model (CFI = 0.936, RMSEA = 0.049). Moderate to strong correlations were found between the DCDDaily-Q-CN performance total score and the DCDQ-R (*r*_s_ = −0.54) and MABC-2 total scores (*r*_s_ = −0.68). The total performance score effectively differentiated between children with DCD and controls (*U* = 9.0, *p* < 0.001), with a cutoff score of 45, demonstrating a sensitivity of 93% (95%CI: 77–99%) and specificity of 90% (95%CI: 74–98%).

**Conclusion:**

The findings support that the DCDDaily-Q-CN is a reliable and valid measure to assess participation and performance in motor-based ADLs and fulfill criterion B of the diagnostic criteria for DCD.

## 1 Introduction

Developmental coordination disorder (DCD) is a neurodevelopmental condition characterized by impaired coordination of physical movements that interfere with activities of daily living (ADL) (1). DCD is frequently reported to affect 5–6% of school-age children (2, 3). The Diagnostic and Statistical Manual of Mental Disorders, Fifth Edition (DSM-5) (1) defines DCD by the following four criteria: (A) acquisition and execution of coordinated motor skills is far below age expectations, given the opportunity for skill learning; (B) motor skill difficulties significantly interfere with ADLs and impact academic/school productivity, prevocational and vocational activities, leisure, and play; (C) onset is in the early developmental period; and (D) motor skill difficulties are not better explained by intellectual delay, visual impairment, or other neurological conditions that affect movement. The disorder primarily causes difficulties in performing motor-based daily activities (4) and participating in physical activities such as team sports (5, 6). This may reduce the self-confidence and life satisfaction of those affected (7, 8), ultimately leading to a decrease in long-term participation in social activities (9).

Clinicians and parents have advocated for early identification of children at risk of DCD (10). Valid and accurate standardized assessment scales, including standardized tests and self- or family reported questionnaires, are essential for screening suspected cases, identifying health-related consequences, and gathering diagnostic evidence. For instance, the Movement Assessment Battery for Children Second Edition (MABC-2) (11) and Developmental Coordination Disorder Questionnaire-Revised (DCDQ-R) (12) are the most commonly used instruments to operationalize diagnostic criteria A and B for DCD, respectively. Following the International Classification of Functioning, Disability, and Health-Children and Youth (ICF-CY) framework (13), while evaluating children suspected of having DCD, it is important to employ outcome measures that assess both activity components (such as self-care and mobility) and participation components (such as frequency and involvement of daily activities at home, school, and in the community). This is because family and individual complaints revolve primarily around difficulties in performing daily activities (3).

The DCDDaily-questionnaire (DCDDaliy-Q) is a parental questionnaire that evaluates children’s performance and participation in ADL (14). This tool is the first to comprehensively assess the various ADL difficulties experienced by children with DCD in their everyday lives (3). There is evidence supporting its internal consistency, structural validity, criterion validity, concurrent validity, and discriminative validity (14–17). Percentiles adjusted for age and cut-off scores were established for Dutch children aged 5–8 (14) and Spanish children aged 5–10 (16). Moreover, studies have demonstrated that the DCDDaily-Q is more effective than the currently used questionnaire (DCDQ-R) in predicting DCD (14). Hence, the DCDDaily-Q may be a valuable tool for providing specific information regarding ADL difficulties and participation restrictions related to criterion B of the diagnostic criteria for DCD.

At present, there is no Chinese translation of the DCDDaily-Q. Moreover, population-specific growth references (e.g., reference norms, age and sex-specific growth curves) are absent in the Chinese context. This methodological approach is essential for comprehending regional growth patterns and assists pediatric clinicians in identifying deviations utilizing established references for effective monitoring. Furthermore, sex-specific growth curves provide enhanced understanding of sex-specific development and normative values for clinical referrals. Therefore, this study aimed to (1) cross-culturally adapt the DCDDaily-Q into Chinese (DCDDaily-Q-CN), (2) establish Chinese reference norms and sex-specific growth curves, and (3) assess the psychometric properties of the DCDDaily-Q-CN in Chinese children aged 5–10 years.

## 2 Materials and methods

We followed international guidelines (18, 19) while conducting a cross-cultural adaptation and validation study of the DCDDaily-Q in Chinese population.

### 2.1 Part one: cross-cultural adaptation

**Figure 1:**
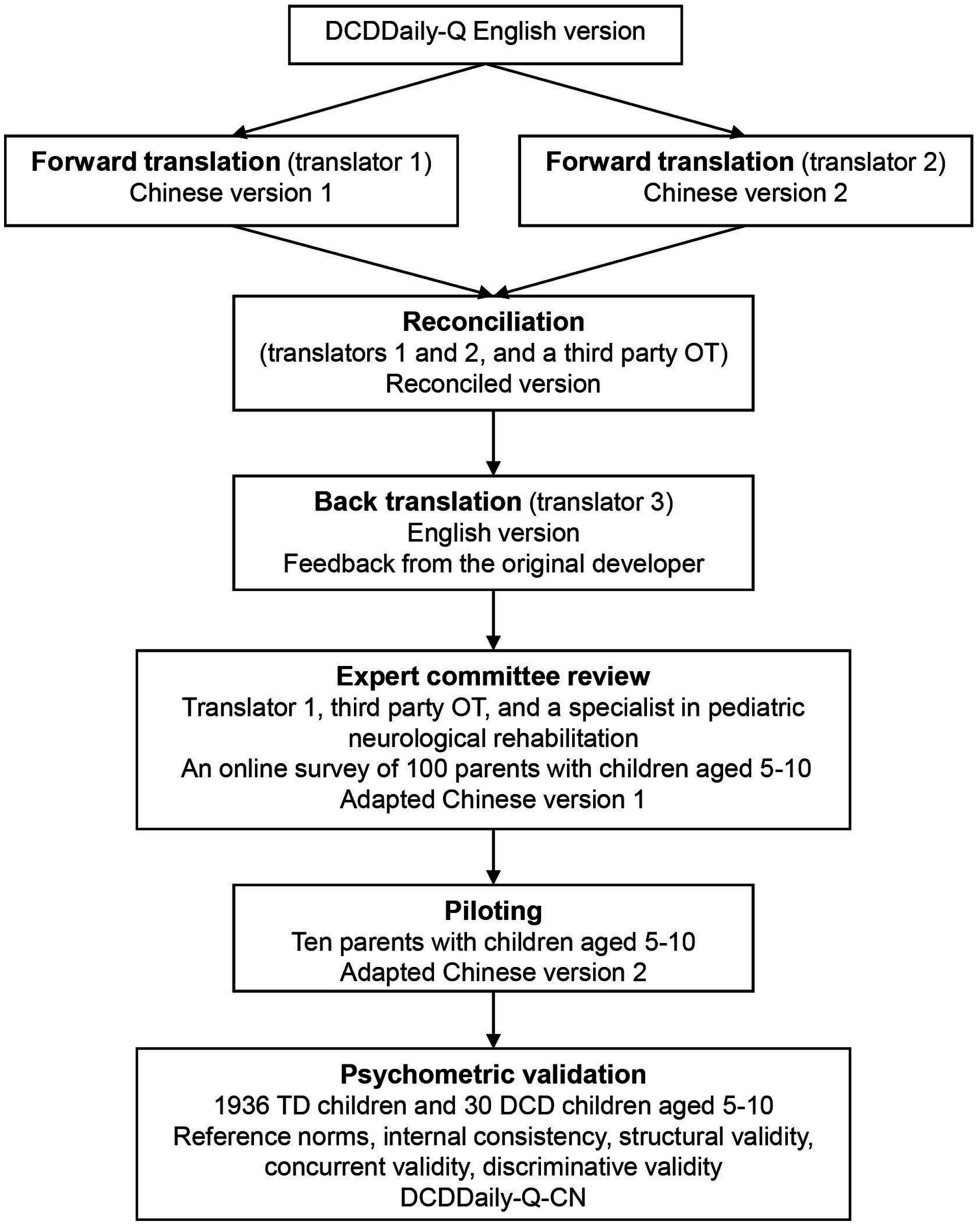
Cross-cultural and psychometric validation process of the DCDDaily questionnaire Chinese version. TD, typically developed; DCD, developmental coordination disorder; OT, occupational therapist.

The original developers granted consent for cross-cultural adaptation and validation of the DCDDaily-Q in Chinese. The adaptation process comprised five essential steps: two independent forward translations, synthesis, back-translation, expert committee review, and piloting with the target population (Figure 1).

Two English speakers independently translated the English version into Chinese; one (T1) was an occupational therapist familiar with DCDDaily-Q and DCD, while the other (T2) was a professional translator unfamiliar with DCDDaily-Q and DCD. The original translators and a third-party occupational therapist (E1), experienced in cross-cultural adaptation research and familiar with DCDDaily-Q, reviewed both translations and discussed all sections of the questionnaire and individual items to produce a unified translation. The criteria for this synthesis are based on source accuracy, cultural appropriateness, grammar, and terminology. A professional translator (T3), unfamiliar with DCDDaily-Q or DCD, back-translated the reconciled Chinese version, which was then sent to the original developer for review. Feedback details were reviewed by an expert committee involving two occupational therapists (T1, E1) and a specialist (E2) in pediatric neurological rehabilitation.

Furthermore, to assess the potential applicability of the DCDDaily-Q in children above 8 years of age, three experts (T1, E1, and E2) examined each activity of the questionnaire to determine its relevance and significance for older children. The experts concluded that the activities were suitable for evaluating daily performance in 9–10-year-old children but not for those exceeding 10 years of age. Consequently, the Chinese version of the DCDDaily-Q was piloted and validated on children aged 5–10 years. Additionally, in order to further evaluate the questionnaire’s suitability in the Chinese cultural context, we conducted an online survey with parents of 100 children aged 5–10. The survey examined how often these children engaged in the 23 activities listed in the DCDDaily-Q and other common daily tasks among Chinese children, as suggested by experts.

The adapted questionnaire, developed from survey results and expert input, was tested on a sample of 10 parents with children aged 5–10 years. These parents came from different geographic regions and had diverse occupational and educational backgrounds. This testing involved individual cognitive debriefing interviews. Piloting is crucial during the cross-cultural adaptation of assessment tools as it aids in pinpointing misunderstandings, concept coverage gaps, and inconsistent interpretations.

### 2.2 Part two: reference norms and psychometric validation

#### 2.2.1 Participants

This study included two groups of children aged 5–10 years: a normative reference group and DCD group.

Norm data were collected from 1936 children aged 5–10 years between March and June 2023. The participants were from 14 randomly selected mainstream schools located in northeast, east, south, and central Shenzhen. With a population of 17 million migrants from across the country, Shenzhen provided a relatively representative sample in terms of geography and ethnicity. None of the participants had been diagnosed with any learning or developmental disorder prior to this study, as confirmed by the schools and parents.

Thirty children with DCD who met the four DSM-5 diagnostic criteria for DCD (1) were recruited through clinical referrals at the Department of Rehabilitation Medicine of Shenzhen Children’s Hospital between March and September 2023. Inclusion criteria: (1) ≤15th percentile on MABC-2 (Criterion A); (2) parental interview confirmed that movement difficulties impacted ADLs (Criterion B); (3) onset was early in the developmental period (Criterion C); and (4) no other condition that may better explain the movement difficulties (Criterion D).

To conduct the validity analysis, a third control group was formed with children from the reference group. This enabled a comparison between children with DCD and those who were typically developed. Children in the reference group were initially selected on the basis of their lack of clinical conditions. From this pool, children were randomly chosen to match the DCD group (*n* = 30) in age (within 1 year), sex, place of birth, region of living, primary carrier and schooling setting, while ensuring that the selection process remained blinded to the outcomes.

#### 2.2.2 Measurements

##### 2.2.2.1 DCDDaily-Q-CN

The DCDDaily-Q is a 23-item parental questionnaire that assesses children’s participation and performance in various ADLs, including self-care and self-maintenance (10 items), fine motor activities (7 items), and gross motor activities (6 items) (14). The DCDDaily-Q was originally developed in the Netherlands and is available in Dutch and English, with translations provided in Spanish (15, 16) and Greek (17). Parents rated their children’s performance on each item compared to an established standard (1 = good, 2 = moderate, 3 = poor). The total performance score ranged from 23 to 69, with higher scores indicating poorer performance. The questionnaire assesses ADL participation in addition to performance using a four-point scale (1 = regularly, 2 = sometimes, 3 = seldom, 4 = not yet/never). The total participation score ranged from 23 to 92, with higher scores indicating lower levels of participation. Parents also indicated whether their children took longer than their peers to learn an activity using a yes/no format (1 = yes; 0 = no).

##### 2.2.2.2 DCDQ-R Chinese version

The DCDQ-R is a widely used tool for evaluating motor coordination in children between the ages of 5 and 15 (12) and is a recommended measurement to fulfill criterion B of the diagnostic criteria for DCD (3). The DCDQ is a parental questionnaire comprising three subscales: control during movement (six items), fine motor/handwriting (four items), and general coordination (five items). The parents were asked to evaluate their children’s performance on a five-point scale. The total DCDQ score ranges from 15 to 75, with higher scores indicating superior performance. This tool has been successfully adapted for use in Chinese children, and its psychometric properties have been demonstrated to be reliable and valid (20).

##### 2.2.2.3 MABC-2 Chinese version

The MABC-2 is a motor skills assessment tool designed for children aged 3–16 years and comprises eight items that evaluate manual dexterity, ball skills, and balance (11). This test is norm-based and is divided into three age bands: 3–6 years, 7–10 years, and 11–16 years. The MABC-2 provides standard scores (range 1–19) and percentiles (range 0–100) for the total scale and each of the three domains. A higher score indicated better performance. The MABC-2 Test is recommended to fulfill diagnostic criterion A for DCD: the total percentile score must be ≤16 or one of the three domain scores ≤5th percentile (3). The tool has been proven valid and reliable in various cultural settings (21), including among Chinese children (22).

#### 2.2.3 Procedures

Ethical approval was obtained from the Ethics Committee for Medical Research of Shenzhen Children’s Hospital (202106202). To collect normative data, the DCDDaily-Q-CN was created as an online survey using RedCap, and distributed to parents or guardians via school intermediation. The parents or guardians accessed the electronic questionnaire by clicking on a link and entering a code. They were instructed to review and sign a written consent prior to beginning the survey and to anonymously and voluntarily complete the questionnaire.

For the DCD group, children were assessed using the MABC-2 test at the rehabilitation center after their parents provided informed consent. Following this, parents were asked to complete online versions of the DCDQ-R and DCDDaily-Q-CN.

#### 2.2.4 Data analysis

Statistical analysis was performed using SPSS version 26.0 (SPSS Inc., Chicago, IL, United States). To determine the impact of age on DCDDaily-Q-CN participation and performance scores, one-way analysis of variance (ANOVA) was performed, and Bonferroni post-hoc tests were used to examine differences between age groups. An independent samples t-test was conducted to assess sex differences in the DCDDaily-Q-CN participation and performance scores. Percentiles for participation and performance scores were calculated. Typically, the 85th and 95th percentiles are used as cut-offs to diagnose DCD in clinical practice and research, respectively (23). However, to provide more information for clinical practice and research, the 80th, 90th, and 96–99th percentiles were also reported. RefCurv was used to create sex-specific percentile curves for performance and participation scores (24).

Structural validity was examined using Confirmatory Factor Analysis (CFA) to test the extent to which the Chinese data fit the initial measurement model (14). Data of the reference group were analyzed with the AMOS 24.0.0 Statistical Package, using maximum likelihood estimation methods. Several goodness-of-fit indices, including the chi-square divided by degrees of freedom (CMIN/DF), root mean square error of approximation (RMSEA), standardized root mean squared residual (SRMR), comparative fit index (CFI), goodness of fit index (GFI), and Tucker–Lewis index (TLI), were used to determine the acceptability of the model. A CMIN/DF value of <5 was considered acceptable. For CFI, GFI, and TLI, values around or above 0.90 are deemed acceptable to good. Ideally, RMSEA and SMRM should be less than or equal to 0.08. Co-varying errors were introduced based on modification indices to improve model fit. This adjustment reflects a nuanced understanding of the data and strengthens the model’s validity by accounting for additional shared variance not captured by the original factor structure alone.

The internal consistency of the DCDDaily-Q-CN was evaluated for the entire scale and for each of the three factors using Cronbach’s alpha coefficient, with values above 0.70 considered acceptable. Additionally, corrected item-total correlations were computed to examine the homogeneity of the items, with values above 0.30 considered acceptable. Concurrent validity was established by computing the Spearman correlation coefficients between the DCDDaily-Q-CN performance and participation scores and the DCDQ-R and MABC-2 total scores for the DCD group. Discriminant validity was established by calculating the differences in participation and performance scores between the DCD and control groups using the Mann–Whitney *U* test. A receiver operating characteristic (ROC) curve was generated to assess the ability of the DCDDaily-Q-CN to differentiate between children with and without DCD based on data from the DCD and control groups. An appropriate cut-off point for the DCDDaily-Q performance score was established to indicate DCD, with high sensitivity and specificity [e.g., at or above 0.80 and 0.90, respectively, along with acceptable 95% confidence intervals (CI)]. The area under the curve (AUC) statistic was calculated to show the likelihood that a child with DCD would have a lower DCDDaily-Q total score than a typically developing child, with a value above 0.80 considered high. In case of missing data in questionnaire measurements, the mean of the remaining answers was used if less than 30% of the answers were missing, otherwise, they were assigned as missing.

## 3 Results

### 3.1 Cross-cultural adaptation

Results of the online cultural suitability survey revealed that less than 2% of Chinese children performed activities such as buttering or cutting sandwiches (Items 1 and 2). Moreover, knives were rarely used as utensils (1%) in China, and only a small percentage had sandwiches for breakfast (27%) or lunch (1%). Following expert advice and parent feedback from the survey, items 1 and 2 were replaced with “poking a straw into a milk carton” and “eating rice with chopsticks” two activities commonly challenging for DCD peers among Chinese children aged 5–10. Additionally, Items 2 to 5 were renumbered to align with the typical routines of Chinese children. Detailed data on item modifications during cross-cultural adaptation processes are presented in Supplementary Table S1. After expert committee review, each item was deemed semantically and conceptually equivalent by at least one expert, eliminating the need for further modifications.

All participants in the pilot trial regarded the items as well written and found the instructions clear. None of the participants experienced any difficulty or misunderstanding nor did they suggest that any section required further modifications or additional examples. Moreover, all activities included in the questionnaire were familiar to the participants. Consequently, the final step of cross-cultural adaptation did not require any modifications to the final version of the DCDDaily-Q-CN. The manual for scoring the DCDDaily-Q-CN is freely available in the Supplementary material.

### 3.2 Reference norms

The RedCap online survey received 2,857 responses, with 921 deemed incomplete (missing over 30% of the answers). Ultimately, 1936 responses were utilized for normative data analysis. The sociodemographic characteristics of the normative sample, DCD group, and control group are presented in Table 1.

**Table 1:** Sociodemographic characteristics and scores on the DCDDaily questionnaire Chinese version of the study groups.

Age group	Reference group	DCD group	Control group
*N*	1936	30	30
Male/female, *n*	1032/904	15/15	15/15
Age, *n*			
5–6 years	757	9	9
7–8 years	632	12	12
9–10 years	547	9	9
Place of birth by province			
Guangdong	1,171	30	30
Hunan	187		
Guangxi	93		
Sichuan	81		
Hubei	76		
Jiangxi	71		
Henan	70		
Chongqing	42		
Anhui	25		
Guizhou	22		
Shaanxi	22		
Fujian	20		
Others^#^	56		
Region of living			
Urban area	1936	30	30
Rural area	0	0	0
Primary carrier, *n*			
Mother	1,480	24	24
Father	425	4	4
Others^*^	31	2	2
Schooling setting, *n*			
Public school	1936	30	30
Private school	0	0	0
DCDDaily-Q-CN Participation, mean (SD)			
All age	36.7 (10.5)	55.6 (9.6)	34.9 (10.3)
Age 5–6 years	38.5 (10.4)	55.2 (7.3)	37.1 (9.3)
Age 7–8 years	36.6 (10.9)	61.2 (8.1)	32.9 (10.2)
Age 9–10 years	34.3 (9.7)	48.7 (9.4)	34.4 (12.0)
DCDDaily-Q-CN Performance, mean (SD)			
All age	33.1 (8.0)	50.5 (4.6)	31.2 (8.1)
Age 5–6 years	34.8 (8.1)	51.8 (1.5)	31.9 (7.9)
Age 7–8 years	32.9 (8.0)	51.8 (6.64)	31.3 (9.3)
Age 9–10 years	30.8 (6.9)	47.6 (0.7)	32.2 (8.0)

DCD, developmental coordination disorder; DCDDaily-Q-CN, DCDDaily questionnaire Chinese version; SD, standard deviation.

# Shandong (*n* = 12), Liaoning (*n* = 12), Gansu (*n* = 9), Zhejiang (*n* = 6), Yunnan (*n* = 6), Jilin (*n* = 5), Inner Mongolia (*n* = 3), Hong Kong (*n* = 3).

* Grandmother (*n* = 18), grandfather (*n* = 5), other relatives (*n* = 8).

**Figure 2:**
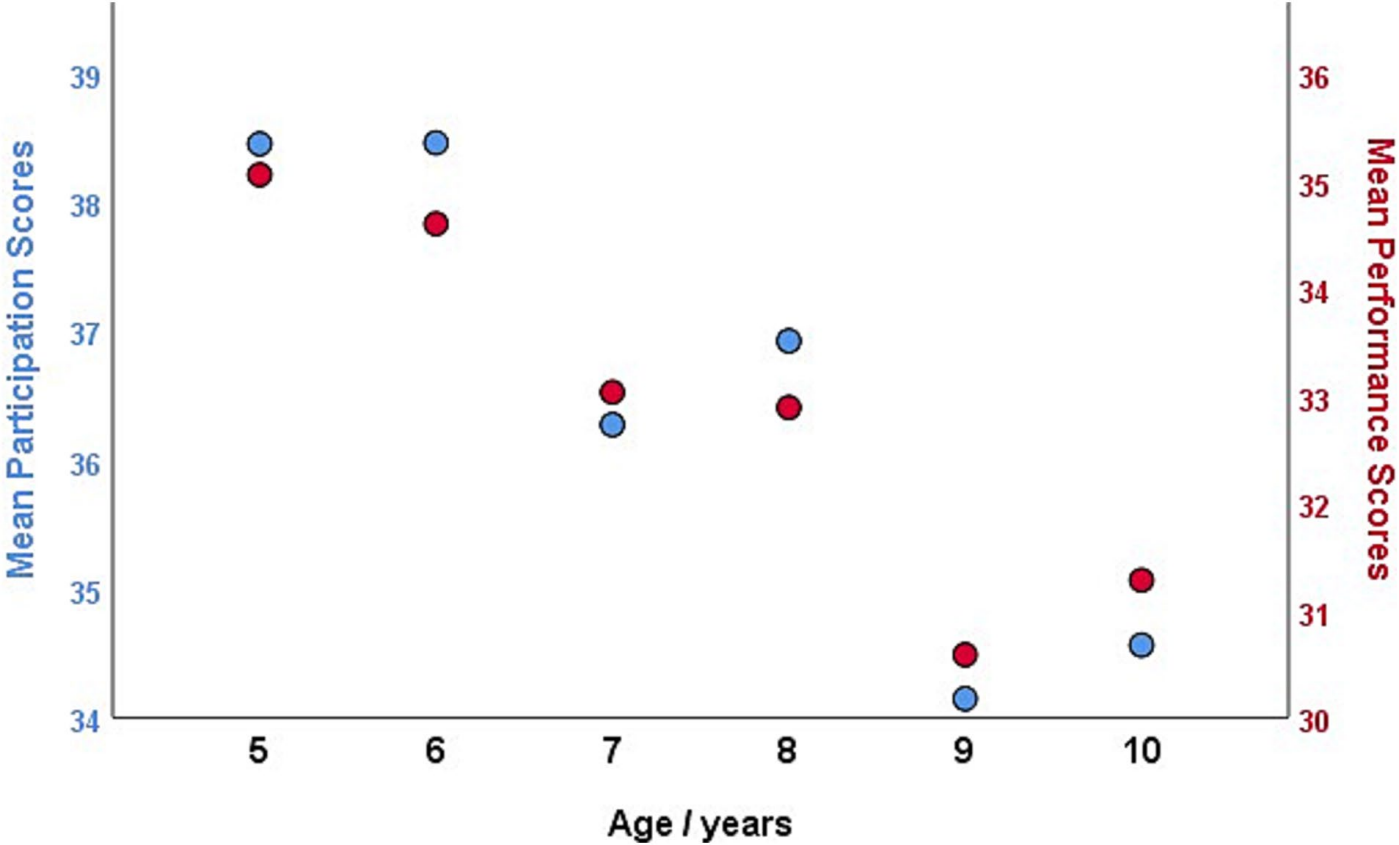
Mean participation and performance total scores of the DCDDaily questionnaire Chinese version by age in the normative sample.

**Figure 3:**
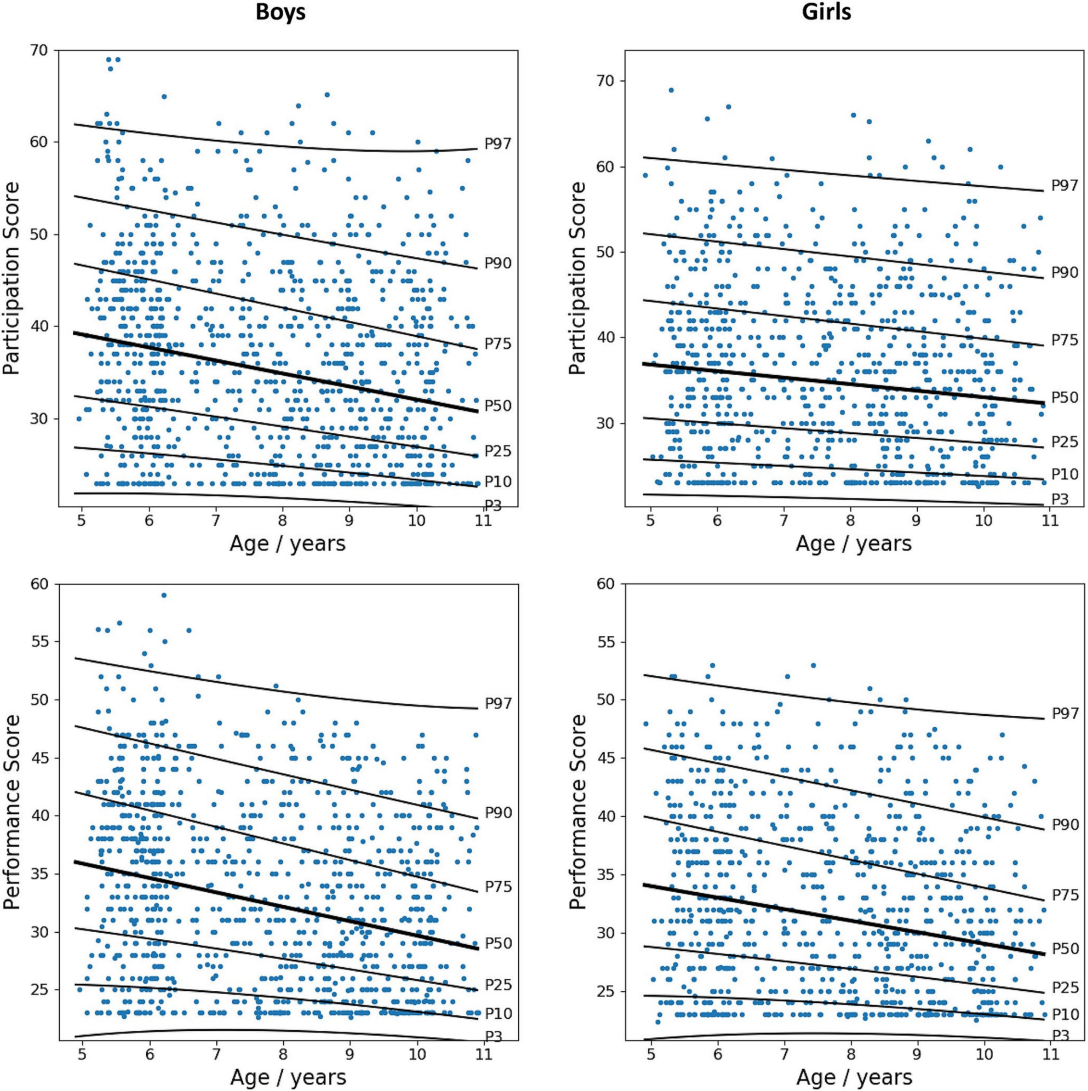
Growth curves for participation and performance total scores of the DCDDaily questionnaire Chinese version in children aged 5–10 years.

ANOVA revealed significant differences in participation [*F*(2,1933) = 21.881, *p* < 0.001] and performance [*F*(2,1933) = 40.406, *p* < 0.001] scores between children aged 5–6 years, 7–8 years, and 9–10 years (see Figure 2). Notably, a significant disparity in performance score was observed between boys (mean [SD] = 33.6 [8.1]) and girls (mean [SD] = 32.5 [7.7]), with boys exhibiting poorer ADL performance (*t* = 2.956, *p* = 0.003). However, no sex-based difference was noted in the participation score (*t* = 1.061, *p* = 0.289). When analyzed by age group, sex differences in both participation and performance scores were observed among children aged 5–6 years. Therefore, percentiles for participation and performance scales of the DCDDaily-Q-CN were calculated separately for children aged 5–6 years, 7–8 years, and 9–10 years, as well as for boys and girls (Table 2). Figure 3 shows the growth curves for ADL participation and performance scores for boys and girls aged 5–10 years.

**Table 2:** Percentiles for participation and performance scores of the DCDDaily-Questionnaire Chinese version across age groups and sex.

DCDDaily-Q-CN	Boys		Girls			
	5–6 years	7–8 years	9–10 years	5–6 years	7–8 years	9–10 years
*n*	393	302	337	317	299	288
Participation score						
p80	47	45	44	46	46	43
p85	49	47	46	50	48	46
p90	52	50	50	52	51	48
p95	58	57	52	56	53	53
p96	58	58	54	57	55	54
p97	60	59	55	58	57	56
p98	62	61	57	60	59	60
p99	68	62	60	65	61	61
Performance score						
p80	43	40	39	41	40	37
p85	45	42	40	43	42	39
p90	47	43	43	45	44	41
p95	49	47	45	48	46	45
p96	50	47	45	48	48	45
p97	52	47	46	49	48	46
p98	54	48	47	49	49	47
p99	56	51	48	52	51	47

DCDDaily-Q-CN, DCDDaily-Questionnaire Chinese version.

The interpretation guidelines for the Chinese percentile cutoff of DCDDaily-Q-CN are presented in Supplementary Table S2. These recommendations were based on the original Dutch DCDDaily-Q manual to enhance consistency in reporting results across different contexts and studies.

### 3.3 Psychometric properties

#### 3.3.1 Internal consistency

The Cronbach’s alpha for the total performance scale of the DCDDaily-Q-CN was 0.917, with subscale values as follows: self-care activities 0.829, fine motor activities 0.837, and gross motor activities, 0.849 (Table 3). All corrected item-total correlations exceeded the accepted threshold of 0.30. Removing any of the DCDDaily-Q-CN items did not alter the internal consistency of the scale.

**Table 3:** Reliability properties of the DCDDaily-Questionnaire Chinese version.

DCDDaily-Q-CN subscale	Items	Cronbach’s alpha	Corrected item-total correlation	Cronbach’s alpha if item deleted
Self-care and self-maintenance	1. Poking a straw into a milk carton	0.829	0.396	0.916
	2. Pouring juice		0.516	0.914
	3. Open a wrapper/package		0.509	0.914
	4. Eating soup with a spoon		0.438	0.915
	5. Eating rice with chopsticks		0.514	0.914
	6. Washing hands		0.485	0.914
	7. Drying oneself after a shower or bath		0.495	0.914
	8. Brushing teeth		0.494	0.914
	9. Handling a key		0.535	0.913
	10. Putting on socks		0.470	0.915
Fine motor activities	11. Writing	0.837	0.591	0.912
	12. Gluing paper using a glue stick		0.645	0.911
	13. Folding paper sheets/slips		0.626	0.912
	14. Coloring a picture		0.569	0.913
	15. Cutting paper using scissors		0.607	0.912
	16. Lego® building		0.520	0.914
	17. Moving pawns (on a board)		0.597	0.912
Gross motor activities	18. Playing hopscotch	0.849	0.581	0.913
	19. Jumping a rope		0.547	0.913
	20. Throwing a tennis ball		0.615	0.912
	21. Catching a ball		0.618	0.912
	22. Kicking a football		0.584	0.913
	23. Playing marbles		0.564	0.913

DCDDaily-Q-CN, DCDDaily-Questionnaire Chinese version.

#### 3.3.2 Structural validity

**Figure 4:**
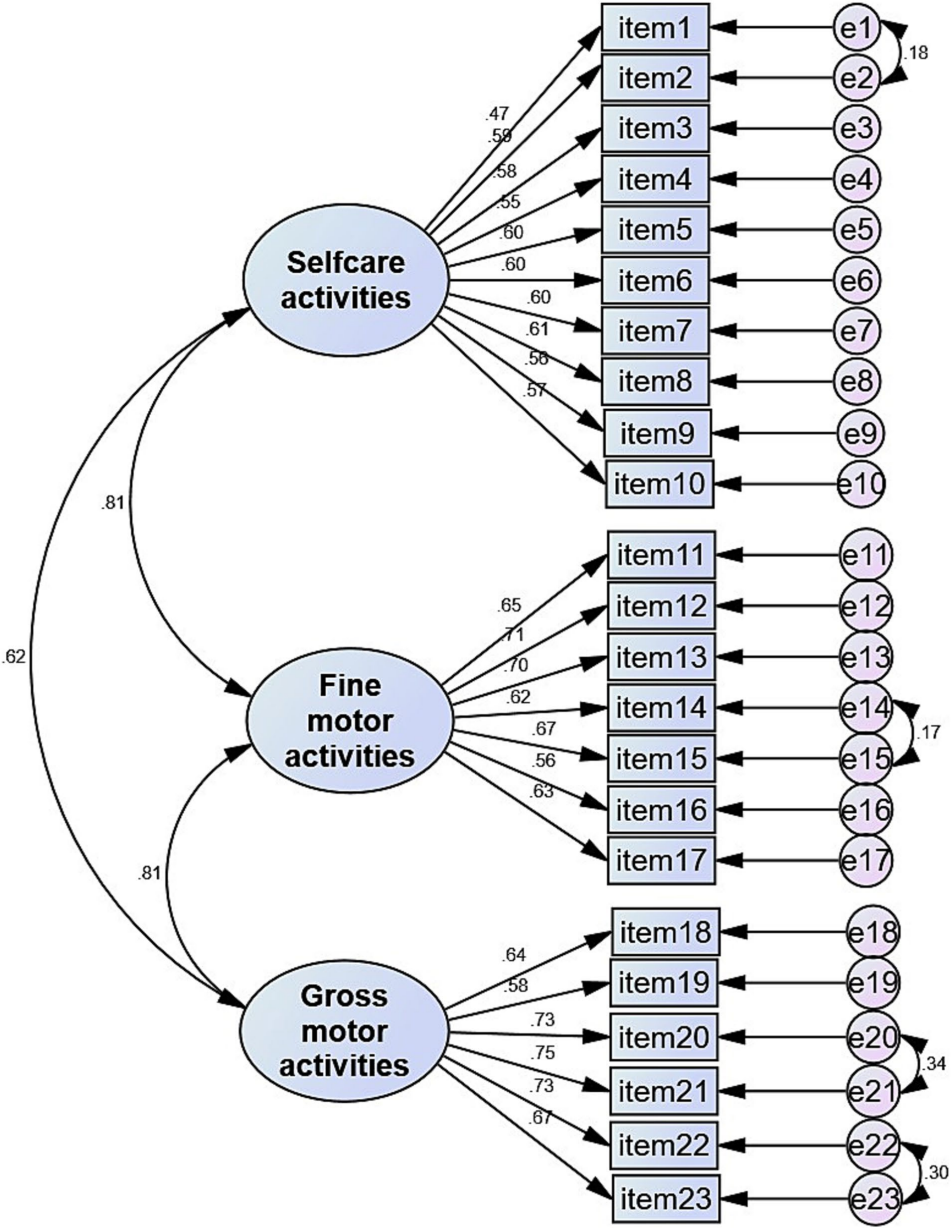
Standardized factor loadings for the best fit measurement model of DCDDaily questionnaire Chinese version.

All the estimated factor loadings found in the CFA were significant at *p* < 0.001, with standardized loadings for each item reaching an acceptable threshold of 0.40 or higher (Figure 4). The modification fit indices suggested a few covariances between the error terms. We allowed four covariations between the error terms on the same factor, and these modifications led to a good degree of model fit. The covariances between errors of items were: item 1 (poking a straw into a milk carton) and item 2 (pouring juice); item 14 (coloring a picture) and item 15 (cutting paper using scissors), item 20 (throwing a tennis ball), item 21 (catching a ball), item 22 (kicking a football), and item 23 (playing marbles). The model fit measures were within their respective common acceptance levels. The original three-factor model (self-care and self-maintenance, fine motor activities, and gross motor activities) yielded a good fit for the data: *χ*^2^ (223) = 1267.01, *p* < 0.001; CMIN/df = 5.682, GFI = 0.942, CFI = 0.936, TLI = 0.928, RMSEA = 0.049, and SMRM = 0.0425.

#### 3.3.3 Concurrent validity

In the DCD group, there were moderate to strong correlations between the DCDDaily-Q-CN performance score and DCDQ-R (*r*_s_ = −0.54, *p* = 0.002), as well as with MABC-2 (*r*_s_ = −0.68, *p* < 0.001).

#### 3.3.4 Discriminative validity

The performance score of the DCDDaily-Q-CN was significantly different between the children with DCD and those in the control group (*U* = 9.0, *p* < 0.001). The ROC curve confirmed that the DCDDaily-Q-CN performance score (AUC = 0.98, *p* < 0.001) effectively differentiated children with and without DCD. A cutoff performance score of 45 demonstrated a sensitivity of 93% (95%CI: 77–99%) and a specificity of 90% (95%CI: 74–98%).

## 4 Discussion

The present study describes the cross-cultural adaptation, reference norms, and psychometric properties of a parental questionnaire designed to evaluate performance and participation in a range of motor-based daily activities. The results highlight the importance of establishing population-based percentiles and cutoffs for evaluating the motor performance and daily participation of children in different cultural contexts. The DCDDaily-Q-CN demonstrated satisfactory internal consistency, structural validity, concurrent validity, and discriminative validity in Chinese children aged 5–10 years.

The DCDDaily-Q was initially developed for the Dutch population (14), with only minor modifications for the Spanish (15) and Greek (17) versions. This is probably due to the common experiences anticipated among European countries, particularly regarding self-care and productive daily activities. However, significant modifications were necessary to the Chinese version of the questionnaire. This was expected, given the notable differences in self-care activities (such as eating habits and utensil usage) between European and Asian cultures. The online survey for cultural suitability showed that Chinese children aged 5–10 rarely engaged in activities such as buttering or cutting sandwiches (items 1 and 2). Moreover, knives and forks are rarely used as utensils, and sandwiches are not commonly eaten for breakfast or lunch within Chinese traditions, aligning with survey results. Therefore, based on expert advice and parent feedback from the survey, we replaced items 1 and 2 with “poking a straw into a milk carton” and “eating rice with chopsticks, “which are common activities for Chinese children aged 5–10 and usually challenging for peers with DCD.

Culturally specific assessment tools are crucial for identifying deviations in developmental patterns, using established references to monitor progress and make informed clinical referrals. The DCDDaily-Q-CN can help identify DCD in other cultural contexts with similar ADL profiles to Chinese, such as Japanese, South Korean, Vietnamese, North Korean, Singaporean, and Malaysian. While activities such as eating, dressing, toileting, bathing, writing, playing and functional mobility are universal experiences; the customs and beliefs surrounding these essential activities for independence vary significantly across regions and ethnicities. Although the culturally adapted DCDDaily-Q-CN may be suitable for these Asian countries; normative reference values based on population still need to be established. Cultural factors should also be taken into account in intervention planning. Through close collaboration with families and a comprehensive understanding of their cultural context, therapists can tailor therapy sessions to address the needs and priorities of both the child and family. For example, Chinese children adhering to traditional eating habits may benefit from practicing self-feeding with chopsticks; conversely, a Chinese child raised in a family with both Eastern and Western customs (e.g., in Hong Kong) may find it more advantageous to learn the use of both chopsticks and Western utensils such as knives and forks. This approach acknowledges cultural diversity while ensuring that therapy goals remain meaningful and relevant to each individual’s unique circumstances.

This study is the first to provide sex-specific growth curves for the DCDdaily-Q, illustrating the development of participation and performance in ADLs among children aged 5 to 10 years. The growth curves demonstrate that participation and performance in ADLs typically increase with age. Older children participated more frequently and performed better than younger children (e.g., 5–6 years old compared to 7–8 years old and 9–10 years old), in line with previous research (14, 16). Motor performance and participation are anticipated to mature with age, as children gain proficiency through experience, especially in self-care ADLs, allowing them to participate in a wider variety of activities as they grow older. Sex differences in ADLs participation were observed across the entire study sample. In particular, boys aged 5 to 6 generally showed lower levels of participation and poorer performance in ADLs than girls of the same age, in contrast to findings from Spanish norms (16) and a Greek sample (17). The impact of sex on motor performance is inconclusive, with some studies showing differences (25, 26) and others not (16, 17). DCD is typically diagnosed more frequently in boys than in girls, with a ratio of 2:1 to 7:1 (3), indicating that boys are more prone to difficulties in motor-based activities at the population level, which may explain the differences observed in the present study sample. Furthermore, cultural factor may also play a significant role in shaping these sex differences in ADL participation and motor performance. Cultural expectations and stereotypes can influence the way boys and girls engage in motor-based ADLs, leading to differences in motor performance. In Chinese culture, parental expectations for girls often emphasize early maturation, independence in self-care activities, proficiency in manual tasks (such as writing, cutting with scissors, sewing, and knitting), and academic excellence. This tendency results in increased opportunities and encouragement for girls in certain motor activities compared to boys, who may experience greater flexibility in their daily activities.

The percentiles and cut-off values for the DCDDaily-Q-CN were established. The participation and performance cutoff scores at the 85th and 95th percentiles for Chinese children were higher than those of Dutch (14) and Spanish (16) children, indicating potentially lower participation levels and poorer performance in ADLs among Chinese children. First, modifications made to items (e.g., replacing “cutting sandwiches” with “eating rice with chopsticks”) on the DCDDaily-Q-CN may have altered the developmental trajectories of ADL acquisition compared to European norms. Consequently, scores should be interpreted with caution when comparing them to non-Chinese populations with different ADL profiles. Second, in the Chinese normative sample, sex-based analyses were conducted with larger sample sizes in each age group (5–6 years, 7–8 years, and 9–10 years), which may account for variations between studies. Third, it is essential to consider that cultural factors significantly influence the development and acquisition of motor-based ADLs. Previous studies have indicated variations in motor performance patterns among children in Europe and Asia. However, the results have been inconsistent: some studies report higher scores in European countries (27), while others demonstrate the opposite trend (28–30). Further investigation is necessary to confirm growth patterns for certain specific types of motor-based ADLs across regions and ethnicities. This underscores the significance of establishing population-specific growth curves and thresholds for diverse ethnic groups when evaluating the risk of DCD in children. The application of criteria derived from other populations, such as Dutch or Spanish cohorts, may lead to inaccurate diagnoses and false positives among Chinese children. In accordance with the Spanish version and conventional diagnostic criteria for DCD (e.g., MABC-2), we propose utilizing the 85th percentile (*z*-score ≈ 1.0) of the performance scale to indicate DCD for criterion B in clinical settings. Conversely, the 95th percentile (*z*-score ≈ 1.5) should be employed in research studies, particularly those of a population-based nature. It is crucial to emphasize that a definitive diagnosis of DCD should only be rendered after a comprehensive evaluation of all four criteria by a multidisciplinary team.

The psychometric properties analysis showed that the DCDDaily-Q-CN is a reliable and valid questionnaire for Chinese children aged 5–10 years. It demonstrated excellent internal consistency across the total and three subscales, similar to the findings from the original (14) and other translated versions (15, 17). The CFA validated the original three-factor theoretical structure (self-care, fine motor, and gross motor activities) of the DCDDaily-Q, which encompasses a wide range of relevant ADLs (14). This is crucial, as parents are intended to complete this questionnaire, necessitating coverage of various occupational areas important to the child (e.g., self-care activities, motor-based school activities) that can be observed by parents (e.g., using cutlery, washing hands, writing, coloring, or playing ball games). The strong correlations between the proposed latent factors additionally confirmed the results of the exploratory factor analysis performed in the original version (14).

Similar to previous research (14, 16), moderate to strong correlations were observed between the DCDDaily-Q-CN total performance score and the DCDQ-R and MABC-2 total scores, indicating sufficient concurrent validity in the Chinese context. The DCDDaily-Q-CN also demonstrated the ability to differentiate between typically developing children and those with DCD: Parents rated the ADL performance of children in the DCD group as significantly poorer than that of the control group. With a cutoff score of 45, the DCDDaily-Q-CN demonstrated a sensitivity of 93% and a specificity of 90%, both meeting the required criteria. This indicates sufficient discriminative validity in identifying children at risk for DCD, confirming the findings of the original validation study (14). Notably, no currently used questionnaire has achieved a satisfactory balance between sensitivity and specificity (at or above 80 and 90%, respectively). For instance, the MABC-2 checklist showed a sensitivity of 41% and specificity of 88% (31), while the DCDQ exhibited a sensitivity of 82% and specificity of 84% (32). It is important to note that each questionnaire evaluates different types of activities related to motor performance during daily living. The DCDQ-R primarily assesses control during movement, fine motor skills, and general coordination, with only one item specifically addressing self-care performance. Similarly, the MABC-2 checklist assesses a wide range of motor skills, such as gross motor coordination, ball skills, fine motor skills, and dynamic balance, with only three items related to self-care performance. On the other hand, the DCDDaily-Q mainly focuses on evaluating self-care ADLs (10/23), along with fine motor activities (7/23) and gross motor activities (6/23). The DCDDaily-Q may better align with criterion B of the diagnostic criteria for DCD by focusing on daily activities such as self-care and motor-based ADLs that are often challenging for the target population (4), thereby showing greater discriminatory ability.

This study has several limitations to consider when interpreting the results. First, while this study included a large sample randomly selected from a city with a significant migrant population across the country to establish reference norms, it is important to note that this sample did not represent the entire population and excluded other ethnic groups. The lack of diverse ethnic representation and the reliance on a single city sample could affect generalizability across other Chinese regions. Subsequent investigations conducted in diverse regions of China, including northern, eastern, and western areas, and encompassing samples from rural populations, will enhance the current study and improve the representativeness of normative data. Second, while we recommend using the 85th and 95th percentiles to indicate DCD in clinical settings and research, it was not feasible to calculate the sensitivity and specificity of the percentiles to identify children with a formal DCD diagnosis. Instead, we provided a general cut-off value that offers a good balance between sensitivity and specificity. The current study included only a small group of clinically confirmed DCD cases, with 15 boys and 15 girls each, which limited the statistical power of ROC analysis based on percentiles. This could result in uncertainty regarding accuracy when utilizing specific percentiles to indicate DCD. Future studies involving larger clinical samples with confirmed DCD diagnoses may help to determine the sensitivity and specificity of the Chinese percentiles in identifying children with DCD. This could improve the utility of DCDDaily-Q-CN in both clinical practice and research.

## 5 Conclusion

This study established sex-specific growth curves for participation and performance development in motor-based daily activities among Chinese children aged 5 to 10 years. Percentiles and cutoff scores of the DCDDaily-Q-CN are provided to indicate children at risk for DCD in clinical settings and research. The questionnaire exhibited satisfactory internal consistency, structural, concurrent, and discriminative validity. Few assessment tools are specifically designed for DCD in China. This study introduced a freely available, reliable, valid, and user-friendly tool to assess motor performance in daily activities and meet criterion B of the diagnostic criteria for DCD. These findings have the potential to enhance the research and clinical investigation of DCD in China and other cultural contexts with similar ADL profiles, thereby improving the recognition of this disorder across a diverse range of global settings.

## Data Availability

The data supporting the findings of this study are available from the corresponding author upon reasonable request.
