# Supplementary material for: Cross-cultural validation and reference norms for the DCDDaily-Q questionnaire Chinese version (DCDDaily-Q-CN): evaluating children’s motor performance in activities of daily living
Source: Front Public Health. 2025 Jan 9;12:1522816. doi: 10.3389/fpubh.2024.1522816 (PMC11754066; doi:10.3389/fpubh.2024.1522816)
Supplement: Supplementary file 1 [file Table_1.DOCX]

Supplementary Material

# Supplementary Tables

**Supplementary Table 1** Details on item modifications of the DCDDaily-Q-CN during cross-cultural adaptation processes

| **DCDDaily-Q**  **Original items** | **DCDDaily-Q-CN**  **Adapted items** | **Item modifications** | **Considerations** |
| --- | --- | --- | --- |
| 1. Buttering a sandwich | 1. Poking a straw into a milk carton | Replacement with “Poking a straw into a milk carton” | - Results of the online cultural suitability survey revealed that less than 2% of Chinese children performed activities such as buttering or cutting sandwiches (Items 1 and 2). Moreover, knives were rarely used as utensils (1%) in China, and only a small percentage had sandwiches for breakfast (27%) or lunch (1%). - Items 2 to 5 were renumbered to align with the typical routines of Chinese children. |
| 2. Cutting a sandwich | 2. Pouring juice | Replacement with “Eating rice with chopsticks”, and renumbered |  |
| 3. Pouring juice | 3. Open a wrapper / package | Renumbered |  |
| 4. Opening a wrapper or package | 4. Eating soup with a spoon | Renumbered |  |
| 5. Eating soup with a spoon | 5. Eating rice with chopsticks | Renumbered |  |
| 6~23 | 6~23 | No modifications |  |

**Supplementary Table 2** Chinese cut-off values and interpretation for the total scores on the “Participation” and “Performance” scales of the DCDDaily-Q-CN

| **DCDDaily-Q-CN** | **5-6 years** | | **7-8 years** | | **9-10 years** | | **Interpretations** |
| --- | --- | --- | --- | --- | --- | --- | --- |
|  | **Boys** | **Girls** | **Boys** | **Girls** | **Boys** | **Girls** |  |
| **Participation** | | | | | | | |
| ≥ p95 | 58 | 56 | 57 | 53 | 52 | 53 | Frequency of participation in ADL is significantly less than peers |
| p86 - p94 | 50~57 | 51~55 | 48~56 | 49~52 | 47~81 | 47~52 | Frequency of participation in ADL is less than peers |
| ≤ p85 | 49 | 50 | 47 | 48 | 46 | 46 | Frequency of participation in ADL is comparable to peers |
| **Performance** | | | | | | | |
| ≥ p95 | 49 | 48 | 47 | 46 | 45 | 45 | Performance of ADL is significantly poorer than peers |
| p86 - p94 | 46~48 | 44~47 | 43~46 | 43~45 | 41~44 | 40~44 | Performance of ADL is poorer than peers |
| 0 - p85 | 45 | 43 | 42 | 42 | 40 | 39 | Performance of ADL is comparable to peers |

DCDDaily-Q-CN, DCDDaily-Q Chinese version; ADL, activities of daily living; p, percentile.
